# The impact of pain on the quality of life of patients with end-stage renal disease undergoing hemodialysis: a multicenter cross-sectional study from Palestine

**DOI:** 10.1186/s12955-021-01686-z

**Published:** 2021-02-02

**Authors:** Aseel F. Samoudi, Maha K. Marzouq, Ahmad M. Samara, Sa’ed H. Zyoud, Samah W. Al-Jabi

**Affiliations:** 1 Department of Medicine, College of Medicine and Health Sciences, An-Najah National University, Nablus, 44839 Palestine; 2 Poison Control and Drug Information Center (PCDIC), College of Medicine and Health Sciences, An-Najah National University, Nablus, 44839 Palestine; 3 Department of Clinical and Community Pharmacy, College of Medicine and Health Sciences, An-Najah National University, Nablus, 44839 Palestine; 4 Clinical Research Centre, An-Najah National University Hospital, Nablus, 44839 Palestine

**Keywords:** End-stage renal disease, Hemodialysis, Quality of life, Pain, Palestine

## Abstract

**Background:**

Chronic kidney disease is considered as a global health problem. Hemodialysis (HD), following renal transplantation, is the most common form of renal replacement therapy. However, HD may impact the quality of life (QOL). Pain is a frequent complaint among this population that also affects their QOL. The purposes of this study were to assess pain and QOL among end-stage renal disease (ESRD) patients on HD and to examine their association.

**Methods:**

This was a multicenter, cross-sectional study that occurred in Palestine between August and November 2018. Brief Pain Inventory and European Quality of Life scale 5 dimensions (EQ-5D) scale, including its European Quality of Life visual analogue scale (EQ-VAS) component, were used to assess pain and QOL, respectively.

**Results:**

A total of 300 participants were included in the final study. The average age of the subjects was 54 ± 16 years. Their median EQ-5D score was 0.68 [0.54–0.88], whereas their median EQ-VAS score was 60 [40–75]. A statistically significant association of pain severity score with EQ-5D score was found (r = − 0.783, *p* < 0.001). The association between pain interference score and EQ-5D score was also found to be statistically significant (r = − 0.868, *p* < 0.001). Similarly, pain severity score was significantly assocsiated with EQ-VAS score (r = − 0.590, *p* < 0.001), the same as was the pain interference score (r = − 0.647, *p* < 0.001). Moreover, age, gender, BMI, employment, educational level, income level, dialysis vintage, previous kidney transplantation, and chronic medication use were all significantly correlated with QOL. Regression analysis showed that patients aged < 60 years (*p* < 0.001), those with lower pain severity scores (*p* = 0.003), and those with lower pain interference scores (*p* < 0.001) had significantly higher QOL scores.

**Conclusions:**

Pain has a significant negative impact on QOL in ESRD patients undergoing HD. The subgroups that were at higher risk included elderly patients, females, those with higher BMI, those without a formal education, those unemployed, those living with low monthly income, smokers, those who have multiple comorbidities, and patients with longer dialysis vintage. Our findings provide reliable data for educators and clinicians working with HD patients.

## Background

In many patients with chronic kidney disease (CKD), their condition progresses to end-stage renal disease (ESRD). Eventually, it involves certain types of renal replacement therapy (RRT) such as renal transplantation, hemodialysis (HD), or peritoneal dialysis [1–3]. The prevalence and incidence of renal failure continue to increase worldwide. According to data collected from 120 countries with dialysis services, approximately 1,900,000 people received renal replacement therapy [4], 1,297,000 (68%) were on HD among those individuals.

In the West Bank in Palestine, the overall number of HD patients continued to increase from 1,119 in 2016 [5] to 1216 in 2017 [6]. About 90.6% of patients requiring RRT were on HD [7]. Despite the vital benefit HD provides, it is still a burdensome method that only postpones imminent death and may result in many clinical side effects, including its negative impact on quality of life (QOL). The frequency and long duration of dialysis sessions limit patients’ independent living and distribute their family and social life [8], which negatively impact patients’ overall health-related quality of life (HRQOL) [9–11]. The index of HRQOL in ESRD patients on HD differs greatly from that of healthy people. This is because patients’ health aims include their ability to lead a secure, independent life [2]. According to a 2018 study, ESRD was reported as the sixth leading source of disease burden in Palestine, accounting for 3.6% of disability-adjusted life years resulting from all reported chronic illnesses [12].

Pain, a frequent complaint among ESRD patients, negatively affects the HRQOL and has mental, physical, and social consequences among these patients [13, 14]. According to the International Association for the Study of Pain (IASP), pain is characterized as a hateful sensory or emotional experience that leads to actual harm, including sleep disturbances, symptoms of anxiety and depression, reduced physical activity, impaired interpersonal relationships, and the inability to perform usual activities [15].

The cause of pain has been investigated by many studies [16–20], including a systematic review that reported the presence of vascular access headache and musculoskeletal causes as potential causes of pain. However, about 63% of chronic pain was attributed mostly to osteoarthritis and osteoporosis [16]. Unfortunately, the chronic nature of the pain was also connected to the dialysis procedure itself, peripheral polyneuropathy, peripheral vascular disease, polycystic kidney disease, malignancy, calciphylaxis, and poorly managing of pain [16–20].

Our main aims in the current study were to assess chronic pain in ESRD patients on HD in Palestine, in terms of its severity and interference with activity, as well as to measure the impact it has on the QOL of these patients. We also examined the factors associated with QOL among this group of patients. The findings of this assessment may help healthcare professionals plan and develop pain assessment and management protocols and strategies that aim to address the common signs and symptoms and improve the QOL of this group of patients.

## Methods

### Study design and setting

This was a multicenter, cross-sectional study that was conducted between August and November of 2018 and occurred in the hemodialysis units of five different hospitals: Tulkarem Governmental Hospital, Qalqilya Governmental Hospital, Jenin Governmental Hospital, Tubas Governmental Hospital, and An-Najah National University Hospital (NNUH).

### Study population and sample size

For this study, we included five hemodialysis centers that serve approximately 570 patients in the Northern West Bank [6]. We used the Sample Size Calculator by Raosoft Inc. (http://www.raosoft.com/samplesize.html), with 5% margin of error, 95% confidence interval, a population size of 570. The calculated minimal required sample size was 220. Then we recruited a total of 330 patients by convenient sampling as follows: 74 from Tulkarem Governmental Hospital, 36 from Qalqilya Governmental Hospital, 88 from Jenin Governmental Hospital, 24 from Tubas Governmental Hospital, and 108 NNUH. HD patients were chosen from each kidney dialysis unit using the proportional quota sampling approach to represent the general HD population [21]. The number of patients from each hospital was determined based on the number of patients undergoing dialysis in those dialysis unit. The rationale for this choice was to make the percentage of patients included in each hospital representative of the percentage of patients undergoing dialysis in that hospital from the total number of patients served in all included hospitals. Eventually, participants were selected using a nonprobability convenience sampling technique because of the associated it with saving researchers time, and costs. It was also the easiest method for the accessibility and resources available for the study [22].

### Inclusion and exclusion criteria

The inclusion criteria were that a patient must be aged ≥ 18 years and have been on HD for ≥ 6 months. We excluded any patient who refused to give consent or was unable to proceed with the interview to the end.

### Data collection tool

The patients were evaluated by administering a questionnaire consisting of 4 sections. In the first section, we enquired about demographic and social characteristics including age, gender, occupation, educational level, residency, monthly income level, smoking status, and body mass index (BMI), which was calculated by dividing the subject’s weight in kilograms by their squared height in meters squared. For analysis purposes, smoking status was classified into nonsmoker, light smokers (1–9 cigarettes per day), and smokers (≥ 10 cigarettes per day) [23], whereas BMI was classified into obese weight range (BMI of ≥ 30), overweight range (BMI between 25 and 29.9), normal weight range (BMI between 18.5 and 24.9), and underweight range (BMI of < 18.5) [24].

The second section contained items on clinical and HD-related data, including dialysis vintage (length of time on dialysis treatment in years), frequency per week, duration of sessions, transplantation history (for patients who returned to dialysis after rejection of transplantation), and the number of chronic comorbid illnesses and medications taken chronically.

In the third section, we included the Brief Pain Inventory (BPI) scale, a widely used and accepted pain assessment scale that addresses pain severity, as well as pain interference with daily functions [25]. We obtained permission to use the Arabic BPI-Short Form version of the MD Anderson Cancer Center, which has been previously translated and validated [25, 26]. A Lebanese study found that when the BPI scale was used in a study of Arab patients experiencing pain, the Arabic-language translation showed cultural sensitivity and adequate psychometric properties comparable to the original version [27]. The following topics are assessed in the items on the severity of pain: the worst pain felt during the last 24 h, the least pain felt during the last 24 h, the average pain felt during the last 24 h, and the pain felt at the moment of assessment. Topics covered in the items on the interference of pain with daily functions include: the ability to walk, general mood, ability to work, sleeping quality, social relationships, and the ability to enjoy life generally. BPI also contains items addressing pain sites, pain response to analgesics, and the degree of this response. Responses to the four items on pain severity received a score in the form of a number of points between 0 and 10 for each item, and then a pain severity score was created for each participant by adding up the points received on all four items. Accordingly, pain severity scores ranged from 0 to 40. Likewise, responses on the seven items on pain interference received a score that ranged between 0 and 10 for each item, and a pain interference score was created by adding up the points achieved on all seven items, making the range for this score 0 to 70.

In the fourth and final section of the questionnaire, we included the European Quality of Life Scale 5 dimensions (EQ-5D) assessment tool, which is widely used to evaluate the overall quality of life [28]. EQ-5D is one of the most widely used standardized HRQOL questionnaires available today and has been used in many disorders, including hemodialysis, for clinical assessment in health care [29, 30]. The EQ-5D contains five items that cover the following five domains: self-care, mobility, daily activities, pain and/or discomfort, and anxiety and/or depression [28]. It also contains a visual analog scale—the European Quality of Life visual analogue scale (EQ-VAS)—that allows subjects to report on their perception of their health and life quality by answering items on a grading system that ranges from 0 to 100, with 0 representing the worst possible health situation and 100 representing the best possible health situation [31]. We obtained approval to use the Arabic version of EQ-5D in this study from the Euro-QOL Research Foundation (registered ID: 28409). The principal investigator in the current study has previously published a guide on the use of the EQ-5D scale, as well as other projects that employed this tool [32–34]. The EQ-5D score indexing that was used here has been illustrated elsewhere [34–38] and used the EQ-5D-5L Crosswalk Index Calculator [39] based on the scoring algorithm of the UK general population. Two academic experts specialized in clinical pharmacy, who also have expertise in research on QOL and statistical analysis, reviewed the questionnaire components in terms of face validity and content validity, as well as clinical accuracy. Face-to-face interviews were implemented by trained researchers for data collection. The questionnaire was pilot-tested on 15 subjects, which were not included in the final study, to determine its comprehensibility and clarity, and the time needed to complete it.

### Data analysis

SPSS version 21 was used in this study for data analysis. Categorical variables were described in the form of frequencies and percentages, and continuous variables were described in the form of mean and standard deviations or medians and interquartile ranges. For continuous variables, normality was also tested using the Kolmogorov–Smirnov test. The associations of the participants’ characteristics with their scores on the used scales were calculated by Kruskal–Wallis H and Mann–Whitney U tests. We also used the Spearman correlation coefficient to test the association degree between participants’ scores on the used scales. *p* values of < 0.05 were accepted as significant. Additionally, we used multiple linear regression analysis to determine factors that were independently associated with quality of life. We entered into the regression model all variables (demographic and clinical, as well as pain severity and interference) that were significantly associated with quality of life in the bivariate analysis. Cronbach’s alpha was used to check the internal consistency reliability of the scale.

## Results

### Sociodemographic and clinical characteristics

A total of 330 ESRD patients undergoing HD were contacted to participate in this study; 300 of them decided to participate, whereas the rest refused to take part in this study, accounting for a response rate of 90.9 percent. Demographic and clinical variables are presented in detail in Table 1. The participants’ mean age in years was 54 ± 16, with 60.7% of them being < 60 years old. Slightly more than half of the participants (55.3%) were males, and 73.3% of all subjects were married. Subjects living in villages accounted for 58.3% of the sample, and those living with their families accounted for 93.7%. The majority of participants (59.3%) lived in a household with an income of < 2000 NIS (1 NIS = 0.31 US Dollars) per month.

**Table 1:** Participants’ European Quality of Life scale five dimensions (EQ-5D) scores by demographic and clinical characteristics

Variable	Frequency (%);	Median [Q1–Q3]	*p* value^a^
	N = 300		
*Age category (years)*			
Less than 60	182 (60.7)	0.77 [0.64–1.00]	< 0.001^b^
60 or more	118 (39.3)	0.59 [0.17–0.72]	
*Gender*			
Male	166 (55.3)	0.72 [0.58–0.88]	0.003^b^
Female	134 (44.7)	0.65 [0.43–0.77]	
*Body mass index category*			
Underweight	12 (4.0)	1.00 [0.67–1.00]	< 0.001^c^
Healthy	96 (32.0)	0.77 [0.63–1.00]	
Overweight	113 (37.7)	0.71 [0.41–0.88]	
Obese	79 (26.3)	0.64 [0.43–0.68]	
*Residency*			
Refugee camp	30 (10.0)	0.65 [0.37–0.81]	0.141^a^
Village	175 (58.3)	0.71 [0.56–0.88]	
City	95 (31.7)	0.68 [0.40–0.85]	
*Living arrangement*			
Alone	19 (6.3)	0.71 [0.55–0.77]	0.895^b^
With family	281 (93.7)	0.68 [0.54–0.88]	
*Educational level*			
No formal education	14 (4.7)	0.49 [0.34–0.65]	< 0.001^c^
Primary school	62 (20.7)	0.62 [0.31–0.71]	
Secondary school	81 (27.0)	0.68 [0.54–0.88]	
High school	67 (22.3)	0.77 [0.65–1.00]	
Graduated	76 (25.3)	0.77 [0.63–0.97]	
*Social status*			
Married	220 (73.3)	0.70 [0.57–0.87]	0.291^b^
Single, divorced, or widowed	80 (26.7)	0.67 [0.17–1.00]	
*Employment*			
Unemployed	235 (78.3)	0.68 [0.54–0.80]	< 0.001^b^
Employed	65 (21.7)	0.88 [0.64–1.00]	
*Household monthly income*			
< 2000 NIS	178 (59.3)	0.67 [0.43–0.81]	< 0.001^c^
2000–4999 NIS	109 (36 .3)	0.73 [0.57–1.00]	
≥ 5000 NIS	13 (4.3)	0.88 [0.73–1.00]	
*Dialysis vintage (years)*			
< 4	196 (65.3)	0.71 [0.55–0.88]	0.037^b^
≥ 4	104 (34.7)	0.65 [0.46–0.84]	
*Dialysis sessions per week*			
< 3	21 (7.0)	0.71 [0.62–0.81]	0.591^c^
3	276 (92.0)	0.68 [0.54–0.88]	
≥ 4	3 (1.0)	0.68 [0.15-N/A]	
*Dialysis session duration (hours)*			
< 4	258 (86.0)	0.68 [0.54–0.88]	0.629^b^
≥ 4	42 (14.0)	0.68 [0.63–0.82]	
*Previous kidney transplantation*			
Yes	13 (4.3)	1.00 [0.68–1.00]	0.040^b^
No	287 (95.7)	0.68 [0.54–0.88]	
Known cause for ESRD			0.454^b^
Yes	281 (93.7)	0.70 [0.54–0.88]	
No	19 (6.3)	0.68 [0.40–0.88]	
Chronic diseases category	30 (10)		
None	77 (25.7)	0.86 [0.67–1.00]	< 0.001^c^
1	86 ( 28.7)	0.77 [0.68–1.00]	
2	107 (35.7)	0.71 [0.58–0.88]	
≥ 3		0.57 [0.17–0.73]	
*Chronic medications category*			
< 4	10 (3.3)	0.68 [0.58–0.86]	0.868^b^
≥ 4	290 (96.7)	0.68 [0.54–0.88]	
Smoking			< 0.001^c^
Non smoker	218 (72.7)		
Light smoker	48 (16)		
Smoker	34 (11.3)	0.65 [0.51–0.81]	
		0.75 [0.65–0.88]	
		0.88 [0.78–1.00]	
Pain severity			< 0.001^c^
Mild	219 (73)	0.77 [0.65– 1.00]	
Moderate	38 (12.7)	0.54 [0.31– 0.60]	
Severe	34 (14.3)	0.36 [0.09–0.32]	
Pain interference			< 0.001^b^
Low	241 (80.3)	0.75 [0.64–0.88]	
High	59 (19.7)	0.08 [0.06–0.52]	

ESRD, end stage renal disease; NIS, New Israeli shekel (1 NIS = 0.31 US Dollars)

^a^Bold values denote statistical significance at the *p* < 0.05 level

^b^Mann–Whitney U test

^c^Kruskal–Wallis test

Regarding HD-related characteristics, 65.3% had been on HD for less than four years, 92.0% received HD three times per week, and 86.0% received HD sessions for a duration of less than four hours per session. Only 4.3% reported a history of kidney transplantation. Most subjects had less than three other chronic illnesses, with only 35.7% living with ≥ 3 chronic comorbid diseases. However, 96.7% were on ≥ 4 chronically used medications and 82% of all subjects reported taking their medication by themselves.

### Brief Pain Inventory score

The means for pain severity score and pain interference score as found in our study were 10.55 ± 10.62 and 19.41 ± 18.51, respectively, whereas the medians for these scores were 8 [0.00–17.75] and 16.5 [0.00–30], respectively. Additionally, the interreliability indices for the pain severity and pain interference subscales were good, with a Cronbach’s alpha of 0.947 and 0.963, respectively.

### EQ-5D and EQ-VAS scores

**Fig. 1:**
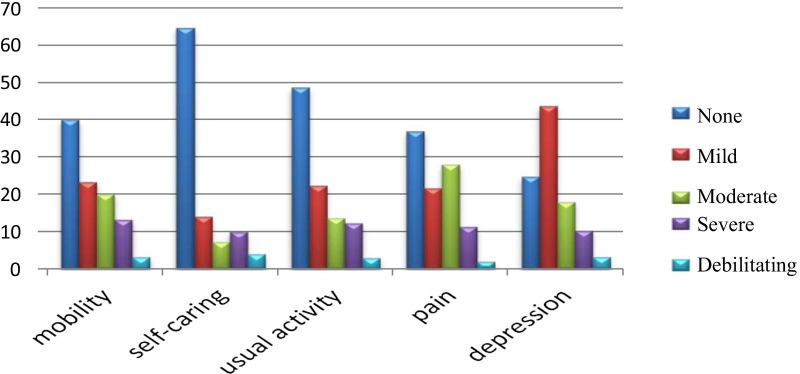
Distribution of quality of life indices in different domains of the European Quality of Life scale 5 dimensions (EQ-5D)

Our sample's median EQ-5D score was 0.68, with an interquartile range of [0.51–0.84], whereas the mean EQ-5D was 0.63 with a standard deviation of 0.34. Internal consistency was measured by Cronbach’s alpha, and the result was 0.936. On the other hand, the median EQ-VAS score was 60.00 [60.00–80.00], whereas the mean EQ-VAS score was 57.53 ± 25.09. We found that the distribution of patients who reported the worst health status across all dimensions of EQ-5D was as follows: mobility 3.3%, usual activity 3%, self-care 4%, pain and/or discomfort 2%, and depression and/or anxiety 3.3%, whereas the percentage of patients who reported the worst in any dimension was 2% (Fig. 1).

### Univariate and multiple linear regression analysis

Table 1 shows the significant differences (*p* < 0.05) between participants according to age, gender, BMI, educational level, employment, monthly income, dialysis vintage, history of kidney transplantation, chronic medication use, and smoking history in association with EQ-5D scores. Table 1 presents these results in detail. We also found significant negative correlations for both pain interference score (r of -0.868, *p* < 0.001) and pain severity score (r of -0.783, *p* < 0.001) with the EQ-5D score.

Table 2 shows the results of the relationship between participants’ characteristics and their EQ-VAS scores. Age, BMI status, educational level, work, monthly income level, dialysis vintage, chronic diseases, and chronic medications were all significantly correlated with EQ-VAS score. We also found a significant negative association between EQ-VAS score and both pain severity score (r = − 0.590, *p* < 0.001) and pain interference score (r = − 0.647, *p* < 0.001), as well as a significant positive association between the EQ-5D score and EQ-VAS score (r = 0.742, *p* < 0.001). The results of the linear regression analysis showed that patients aged < 60 years (*p* < 0.001), with lower pain severity scores (*p* = 0.003), or with lower pain interference scores (*p* < 0.001) had significantly higher QOL scores. Table 3 summarizes the results of multiple linear regression analysis of factors associated with QOL.

**Table 2:** Participants’ European Quality of Life visual analogue scores by sociodemographic and clinical characteristics

Variable	Frequency (%); N = 300	Median [Q1-Q3]	*P* value^a^
*Age category (years)*			
Less than 60	182 (60.7)	70 [50–80]	**< 0.001**^b^
60 or more	118 (39.3)	50 [29–70]	
*Gender*			
Male	166 (35.3)	60 [50–75]	0.429^b^
Female	134 (44.7)	66 [40–77]	
*Body mass index category*			
Underweight	12 (4.0)	70 [25–88]	**0.003**^c^
Healthy	96 (32.0)	70 [50–80]	
Overweight	113 (37.7)	60 [40–80]	
Obese	79 (26.3)	50 [35–70]	
*Residency*			
Refugee camp	30 (10.0)	75 [38–81]	0.141^c^
Village	175 (58.3)	63 [50–75]	
City	95 (31.7)	60 [30–75]	
*Living arrangement*			
Alone	19 (6.3)	70 [50–75]	0.269^b^
With family	281 (93.7)	60 [40–75]	
*Educational level*			
No formal education	14 (4.7)	48 [18–71]	** < 0.001**^c^
Primary school	62 (20.7)	50 [30–70]	
Secondary school	81 (27.0)	60 [40–77]	
High school	67 (22.3)	60 [50–75]	
Graduated	76 (25.3)	70 [55–84]	
*Social status*			
Married	220 (73.3)	60 [44–75]	0.729^b^
Single, divorced, or widowed	80 (26.7)	70 [31.2–80]	
*Employment*			
Unemployed	235 (78.3)	60 [40–70]	** < 0.001**^b^
Employed	65 (21.7)	75 [60–87]	
*Household monthly income*			
< 2000 NIS	178 (59.3)	60 [39–75]	**0.001**^c^
2000–4999 NIS	109 (36 .3)	65 [48–80]	
> 5000 NIS	13 (4.3)	78 [70–85]	
*Dialysis vintage(years)*			
< 4	196 (65.3)	65 [41–78]	**0.040**^b^
≥ 4	104 (34.7)	58 [31–75]	
*Dialysis sessions per week*			
< 3	21 (7.0)	65 [53–73]	0.787^c^
3	276 (92.0)	60 [40–77]	
≥ 4	3 (1.0)	75 [50-N/A]	
*Dialysis session duration (hours)*			
< 4	258 (86.0)	65 [40–77]	0.740^b^
≥ 4	42 (14.0)	60 [50–71]	
*Previous kidney transplantation*			
Yes	13 (4.3)	80 [40–78]	0.258^b^
No	287 (95.7)	60 [40–75]	
*Known cause for ESRD*			
Yes	281 (93.7)	65 [40–78]	0.439^b^
No	19 (6.3)	60 [40–70]	
*Chronic diseases category*			
None	30 (10.0)	75 [50–89]	**< 0.001**^c^
1	77 (25.7)	70 [50–80]	
2	86 (28.7)	65 [50–79]	
≥ 3	107 (35.7)	50 [20–70]	
*Chronic medications category*			
< 4	10 (3.3)	50 [28–59]	**0.037**^b^
≥ 4	290 (96.7)	65 [40–77]	
*Smoking*			
Nonsmoker	218 (72.7)	65 [40–75]	0.439^c^
Light smoker	48 (16.0)	55 [50–80]	
Smoker	34 (11.3)	68 [50–82]	
*Pain severity*			
Mild	219 (73.0)	70 [50–80]	**< 0.001**^c^
Moderate	38 (12.7)	50 [35–61]	
Severe	34 (14.3)	12 [10–40]	
*Pain interference*			
Low	241 (80.3)	70 [50–80]	**< 0.001**^b^
High	59 (19.7)	20 [10–50]	

ESRD, end stage renal disease; NIS, New Israeli shekel (1 NIS = 0.31 US Dollars)

^a^Bold values denote statistical significance at the *p* < 0.05 level

^b^Mann–Whitney U test

^c^Kruskal–Wallis test

**Table 3:** Multiple linear regression analysis of association between participants’ characteristics and their quality of life (EQ-5D-Score)

Variables	Unstandardized coefficients		Standardized coefficients	T	*p* value*	95.0% confidence interval for B		Collinearity statistics
	B	Std. error	Beta			Lower bound	Upper bound	VIF
(Constant)	1.1	0.13		8.77	< 0.001	0.85	1.35	
Age	− 0.1	0.02	− 0.15	− 4.83	**< 0.001**	− 0.15	− 0.06	1.26
Gender	0	0.02	0	− 0.07	0.941	− 0.04	0.04	1.27
BMI	− 0.01	0.01	− 0.01	− 0.46	0.644	− 0.03	0.02	1.19
Educational level	0	0.01	− 0.02	− 0.56	0.578	− 0.02	0.01	1.31
Occupational status	0.02	0.03	0.03	0.79	0.43	− 0.03	0.07	1.43
Income	0.02	0.02	0.04	1.32	0.189	− 0.01	0.06	1.25
Dialysis vintage	− 0.01	0.02	− 0.02	− 0.74	0.462	− 0.05	0.02	1.08
Previous kidney transplantation	− 0.02	0.05	− 0.01	− 0.35	0.723	− 0.11	0.08	1.08
Number of chronic diseases	0	0.01	0	− 0.14	0.886	− 0.02	0.02	1.38
Pain severity score	− 0.01	0	− 0.16	− 2.98	**0.003**	− 0.01	0	2.03
Pain interference score	− 0.01	0	− 0.67	− 11.78	**< 0.001**	− 0.01	− 0.01	2.27

^*^Bold values denote statistical significance at the *p* < 0.05 level

## Discussion

The current study administered a thorough analysis of QOL in ESRD patients on HD in the Northern West Bank in Palestine. We used the EQ-5D scale and its EQ-VAS component to measure QOL and the Brief Pain Inventory scale to assess chronic pain symptoms. Many demographic and clinical factors can affect QOL. In this study, demographic factors correlated with lower QOL included old age, high BMI, female sex, residing in a camp, having no formal education, being unemployed, and living with a low income.

Patients with ESRD receiving maintenance HD suffer from many physical symptoms, including pain, which affects the QOL [40]. Many studies used the EQ-5D scale to assess QOL among ESRD patients [40–47]. Since this tool measures the improvement and deterioration in QOL, it can be used to evaluate QOL and subsequently test the effectiveness of interventions to improve QOL among ESRD patients on HD.

The participants’ mean EQ-5D score was 0.63 ± 0.34. This result is close to results from previous studies that occurred in Singaporean, Thailand, Switzerland, and the UK, which reported EQ-5D scores of 0.704 ± 0.199 [48], 0.65 ± 0.23 [49], 0.62 ± 0.30 [50], and 0.60 ± 0.28, respectively [51]. However, our study's mean EQ-5D score was higher than that of two previous studies conducted in Palestine [21, 34], which may be due to our use of a convenience sampling technique. Among ESRD patients undergoing HD, several socioeconomic and healthcare system-related factors may affect HRQOL. Differences in the sociodemographic and clinical characteristics of HD patients, such as age, prevalence of comorbid diseases, and dialysis vintage, could explain some of these differences in the EQ-5D scores [34, 52].

The association that we found between advanced age and lower QOL is similar to the findings of previous studies from Iran [53] and Korea [54], as well as other studies that were conducted in Palestine [21, 32, 34]. One probable explanation is that older patients tend to withdraw more from social life with illness progression and become physically less active, which reflects negatively on their QOL [55].

We also found that gender and BMI were significantly associated with QOL, with females and those with higher BMI having worse QOL. It has been suggested in previous studies that higher levels of physical inactivity among females [53, 56], with the resulting higher rates of stress and depression, could be a contributing factor [38, 57–59], which could also have a role in explaining the findings of the current study as well, especially considering the established association between BMI and QOL [60].

Moreover, our results showed that having a lower level of education, being unemployed, and living with a low income negatively impact the QOL, similar to results from previous studies [9, 61, 62]. This may be due to differences in the ability to understand the illness's nature and how it can affect QOL [32, 53]. Additionally, other studies have concluded that a higher educational level is an important factor in being more compliant with treatment [63–66] and emphasized the positive effect of having a higher income on the QOL in terms of the ability to obtain treatment and other health services [46, 67].

In addition to that, our study showed that some clinical and HD-related characteristics were associated with QOL, including time since starting dialyzing, history of kidney transplantation, level of knowledge about the cause of their disease, smoking, and the number of other comorbid diseases. For example, we found a longer duration since starting on dialysis was significantly associated with lower QOL, which is similar to the findings from other studies [43, 45]. Moreover, patients with a history of kidney transplantation achieved higher QOL scores, a finding that has also been reported in other studies [68–70]. Living with three or more comorbid diseases was significantly associated with lower QOL as well. We did not test this association with specific comorbidities in the current study, but other studies have reported a similar association with diabetes mellitus and hypertension [38, 41, 48, 65, 71].

The mean EQ-VAS score for our participants was somewhat higher than the mean score reported by a similar survey in Thailand [49]. In our study, old age, obese weight range, having no formal education, unemployed, and living with a lower income were all associated with lower EQ-VAS scores. Some clinical characteristics were also associated with lower EQ-VAS scores, including having been on HD for more than four years and living with more than three comorbidities. These results were in accordance with that of other similar studies [21, 34].

We also found a positive association between EQ-5D score and EQ-VAS, which indicates that using more than one scale may increase the accuracy of the result for QOL assessment. Finally, there was a significant negative association between the severity of pain and interference in daily life, on the one hand, and QOL on the other. Many studies have reported this correlation, which emphasizes the need to pay more attention to any complaints of pain and adopt an early management protocol to decrease the negative complaints on QOL [42, 44, 72, 73]. Additional studies are required to explore possible pain management interventions and test the effects of such interventions on QOL. For example, one recent study that was conducted in Turkey found that progressive relaxation exercises may decrease pain and improve QOL among patients undergoing HD treatment [74].

### Strengths and limitations

Our study’s strengths include its multicenter setting and its relatively large sample size. This was also the first study in the West Bank, Palestine, to examine the effect of pain and other social, demographic, and clinical characteristics on QOL among ESRD patients on HD. Furthermore, the current study used the EQ-5D and BPI scales, which are widely accepted assessment tools to study QOL and pain symptoms, respectively. However, this study had some limitations, including its cross-sectional design, which left any speculation on causality unreliable. Moreover, using the convenience sampling technique may have reduced the generalizability of the results of this study to other HD patients. Finally, even though the scale we used to assess QOL is a widely used and accepted tool, using other scales in addition to or instead of it might have yielded additional data that would have helped us better characterize QOL in our target population.

## Conclusions

We found that pain symptoms have a significant negative impact on life quality among ESRD patients who were on HD treatment. The subgroups of this population that were at higher risk of having worse QOL included elderly patients, females, those with obesity, weight range, BMI, those without formal education, unemployed, those living with low monthly income levels, smokers, and those who have multiple comorbidities, as well as patients with longer dialysis vintage. Our findings provide a source of reliable data for educational institutions, as well as clinicians working with ESRD patients and managing their symptoms, including chronic pain to achieve a high level of pain relief. Healthcare professionals and policymakers should give special attention to chronic pain and overall QOL in patients with HD, especially those with ESRD. Standard guidelines for the management and follow-up of chronic pain and life quality should also be implemented, and programs to increase the public awareness of the causes of ESRD should be designed to decrease patient suffering and healthcare costs.

## Data Availability

The datasets used for the current study are available from the corresponding authors (samahjabi@yahoo.com; saedzyoud@yahoo.com) upon request.

## Abbreviations

CKD: Chronic kidney disease
ESRD: End-stage renal disease
HD: Hemodialysis
QOL: Quality of life
NNUH: An-Najah National University Hospital
BMI: Body mass index
NIS: New Israeli shekel
BPI: Brief Pain Inventory
EQ-5D: European Quality of Life scale 5 dimensions
EQ-5D-5L: European Quality of Life scale 5 dimensions 5 levels
EQ-VAS: European Quality of Visual Analogue Scale
IRB: Institutional Review Board

## References

[1] Swarnalatha G, Ram R, Prasad N, Dakshinamurty KV (2011). End-stage renal disease patients on hemodialysis: a study from a tertiary care center in a developing country. Hemodial Int.

[2] KDIGO consortium: Chapter 1: Definition and classification of CKD. *Kidney Int Suppl (2011)* 2013;3(1):19–62. 10.1038/kisup.2012.64 PMC4089693 25018975

[3] Ruwanpathirana T, Senanayake S, Gunawardana N, Munasinghe A, Ginige S, Gamage D, Amarasekara J, Lokuketagoda B, Chulasiri P, Amunugama S (2019). Prevalence and risk factors for impaired kidney function in the district of Anuradhapura, Sri Lanka: a cross-sectional population-representative survey in those at risk of chronic kidney disease of unknown aetiology. BMC Public Health.

[4] Hall YN, Chertow GM (2002). End stage renal disease. BMJ Clin Evid.

[5] Ministry of Health, Palestinian Health Information Center. Health Status, Palestine, 2015. 2016. http://www.moh.ps/Content/Books/NWNJXX7RJ92Bn4f5EGYiH43a2tjAAzKBnseGnEUCaqWqYZndsbCcPy_JQWguvkHTR4Xk4zUpdT45ooWxH11BhIbVAxwpGWy2wiwHdGcM5K7aZ.pdf. Accessed 12 Nov 2019.

[6] Ministry of Health, Palestinian Health Information Center. Health Annual Report, Palestine 2017. 2018. https://www.site.moh.ps/Content/Books/SqKowAFT5W8X4JiBEq9G7oxhDAj5kMzdy3ptPxXOpqK2DzurAO7A9f_1NTofLKiqyKkOVOIhEOjpD91OKHIovSZk31vVsJajEEHXkR5vzfy7Q.pdf. Accessed 10 Nov 2019.

[7] Nobahar M, Tamadon MR (2016). Barriers to and facilitators of care for hemodialysis patients; a qualitative study. J Renal Inj Prev.

[8] Ginieri-Coccossis M, Theofilou P, Synodinou C, Tomaras V, Soldatos C (2008). Quality of life, mental health and health beliefs in haemodialysis and peritoneal dialysis patients: investigating differences in early and later years of current treatment. BMC Nephrol.

[9] Wan EY, Chen JY, Choi EP, Wong CK, Chan AK, Chan KH, Lam CL (2015). Patterns of health-related quality of life and associated factors in Chinese patients undergoing haemodialysis. Health Qual Life Outcomes.

[10] Senanayake S, Mahesh PKB, Gunawardena N, Graves N, Kularatna S (2019). Validity and internal consistency of EQ-5D-3L quality of life tool among pre-dialysis patients with chronic kidney disease in Sri Lanka, a lower middle-income country. PLoS ONE.

[11] Kularatna S, Senanayake S, Gunawardena N, Graves N (2019). Comparison of the EQ-5D 3L and the SF-6D (SF-36) contemporaneous utility scores in patients with chronic kidney disease in Sri Lanka: a cross-sectional survey. BMJ Open.

[12] Mosleh M, Dalal K, Aljeesh Y (2018). Burden of chronic diseases in the Palestinian health-care sector using disability-adjusted life-years. Lancet.

[13] Senanayake S, Gunawardena N, Palihawadana P, Bandara P, Haniffa R, Karunarathna R, Kumara P (2017). Symptom burden in chronic kidney disease; a population based cross sectional study. BMC Nephrol.

[14] Senanayake S, Gunawardena N, Palihawadana P, Senanayake S, Karunarathna R, Kumara P, Kularatna S (2020). Health related quality of life in chronic kidney disease; a descriptive study in a rural Sri Lankan community affected by chronic kidney disease. Health Qual Life Outcomes.

[15] Cohen M, Quintner J, van Rysewyk S (2018). Reconsidering the International Association for the Study of Pain definition of pain. Pain Rep.

[16] Brkovic T, Burilovic E, Puljak L (2016). Prevalence and severity of pain in adult end-stage renal disease patients on chronic intermittent hemodialysis: a systematic review. Patient Prefer Adher.

[17] Fleishman TT, Dreiher J, Shvartzman P (2018). Pain in maintenance hemodialysis patients: a multicenter study. J Pain Symptom Manag.

[18] Upadhyay C, Cameron K, Murphy L, Battistella M (2014). Measuring pain in patients undergoing hemodialysis: a review of pain assessment tools. Clin Kidney J.

[19] Sadigova E, Ozkurt S, Yalcin AU (2020). Pain assessment in hemodialysis patients. Cureus.

[20] Kliuk-Ben Bassat O, Brill S, Sharon H (2019). Chronic pain is underestimated and undertreated in dialysis patients: a retrospective case study. Hemodial Int.

[21] Mousa I, Ataba R, Al-ali K, Alkaiyat A (2018). Zyoud SeH: Dialysis-related factors affecting self-efficacy and quality of life in patients on haemodialysis: a cross-sectional study from Palestine. Ren Replace Ther.

[22] Kharusha IK, Sulaiman SS, Samara AM, Al-Jabi SW, Zyoud SH (2020). Assessment of knowledge about first aid methods, diagnosis, and management of snakebite among nursing students: a cross-sectional study from Palestine. Emerg Med Int.

[23] Clair C, Chiolero A, Faeh D, Cornuz J, Marques-Vidal P, Paccaud F, Mooser V, Waeber G, Vollenweider P (2011). Dose-dependent positive association between cigarette smoking, abdominal obesity and body fat: cross-sectional data from a population-based survey. BMC Public Health.

[24] Schmidt M, Johannesdottir SA, Lemeshow S, Lash TL, Ulrichsen SP, Botker HE, Sorensen HT (2013). Obesity in young men, and individual and combined risks of type 2 diabetes, cardiovascular morbidity and death before 55 years of age: a Danish 33-year follow-up study. BMJ Open..

[25] Cleeland CS, Ryan KM (1994). Pain assessment: global use of the Brief Pain Inventory. Ann Acad Med Singap.

[26] Daut RL, Cleeland CS, Flanery RC (1983). Development of the Wisconsin Brief Pain Questionnaire to assess pain in cancer and other diseases. Pain.

[27] Ballout S, Noureddine S, Huijer HA, Kanazi G (2011). Psychometric evaluation of the Arabic brief pain inventory in a sample of Lebanese cancer patients. J Pain Symptom Manag.

[28] Rabin R, de Charro F (2001). EQ-5D: a measure of health status from the EuroQol Group. Ann Med.

[29] Shimizu U, Aoki H, Sakagami M, Akazawa K (2018). Walking ability, anxiety and depression, significantly decrease EuroQol 5-Dimension 5-Level scores in older hemodialysis patients in Japan. Arch Gerontol Geriatr.

[30] Yang F, Devlin N, Luo N (2019). Cost-utility analysis using EQ-5D-5L data: does how the utilities are derived matter?. Value Health.

[31] EQ-5D-5L User Guide: basic information on how to use the EQ-5D-5L instrument.

[32] Zyoud SH, Al-Jabi SW, Sweileh WM, Arandi DA, Dabeek SA, Esawi HH, Atyeh RH, Abu-Ali HA, Sleet YI, Abd-Alfatah BM (2015). Relationship of treatment satisfaction to health-related quality of life among Palestinian patients with type 2 diabetes mellitus: findings from a cross-sectional study. J Clin Transl Endocrinol.

[33] Zyoud SH, Al-Jabi SW, Sweileh WM, Wildali AH, Saleem HM, Aysa HA, Badwan MA, Awang R, Morisky DE (2013). Health-related quality of life associated with treatment adherence in patients with hypertension: a cross-sectional study. Int J Cardiol.

[34] Zyoud SH, Daraghmeh DN, Mezyed DO, Khdeir RL, Sawafta MN, Ayaseh NA, Tabeeb GH, Sweileh WM, Awang R, Al-Jabi SW (2016). Factors affecting quality of life in patients on haemodialysis: a cross-sectional study from Palestine. BMC Nephrol.

[35] Agborsangaya CB, Lahtinen M, Cooke T, Johnson JA (2014). Comparing the EQ-5D 3L and 5L: measurement properties and association with chronic conditions and multimorbidity in the general population. Health Qual Life Outcomes.

[36] Al-Jabi SW, Zyoud SH, Sweileh WM, Wildali AH, Saleem HM, Aysa HA, Badwan MA, Awang R (2014). Assessment of health-related quality of life among hypertensive patients: a cross-sectional study from Palestine. J Public Health.

[37] Al-Jabi SW, Zyoud SH, Sweileh WM, Wildali AH, Saleem HM, Aysa HA, Badwan MA, Awang R (2015). Relationship of treatment satisfaction to health-related quality of life: findings from a cross-sectional survey among hypertensive patients in Palestine. Health Expect.

[38] Saffari M, Pakpour AH, Naderi MK, Koenig HG, Baldacchino DR, Piper CN (2013). Spiritual coping, religiosity and quality of life: a study on Muslim patients undergoing haemodialysis. Nephrology (Carlton).

[39] EuroQol Group. EQ-5D-5L Crosswalk Index Value Calculator. 2016. http://www.euroqol.org/fileadmin/user_upload/Documenten/Excel/Crosswalk_5L/EQ-5D-5L_Crosswalk_Index_Value_Calculator.v2.xls. Accessed 9 June 9 2020.

[40] Ghonemy TA, Allam HM, Elokely AM, Kadry YA, Omar HM (2016). Chronic pain in hemodialysis patients: role of bone mineral metabolism. Alex Med J.

[41] Ayoub AM, Hijjazi KH (2013). Quality of life in dialysis patients from the United Arab Emirates. J Fam Community Med.

[42] Cohen SD, Patel SS, Khetpal P, Peterson RA, Kimmel PL (2007). Pain, sleep disturbance, and quality of life in patients with chronic kidney disease. Clin J Am Soc Nephrol.

[43] Dąbrowska-Bender M, Dykowska G, Żuk W, Milewska M, Staniszewska A (2018). The impact on quality of life of dialysis patients with renal insufficiency. Patient Prefer Adher.

[44] Davison SN, Jhangri GS (2010). Impact of pain and symptom burden on the health-related quality of life of hemodialysis patients. J Pain Symptom Manag.

[45] Gerasimoula K, Lefkothea L, Maria L, Victoria A, Paraskevi T, Maria P (2015). Quality of life in hemodialysis Patients. Mater Sociomed.

[46] Pakpour AH, Saffari M, Yekaninejad MS, Panahi D, Harrison AP, Molsted S (2010). Health-related quality of life in a sample of Iranian patients on hemodialysis. Iran J Kidney Dis.

[47] Zazzeroni L, Pasquinelli G, Nanni E, Cremonini V, Rubbi I (2017). Comparison of quality of life in patients undergoing hemodialysis and peritoneal dialysis: a systematic review and meta-analysis. Kidney Blood Press Res.

[48] Yang F, Griva K, Lau T, Vathsala A, Lee E, Ng HJ, Mooppil N, Foo M, Newman SP, Chia KS (2015). Health-related quality of life of Asian patients with end-stage renal disease (ESRD) in Singapore. Qual Life Res.

[49] Sakthong P, Kasemsup V (2012). Health utility measured with EQ-5D in Thai patients undergoing peritoneal dialysis. Value Health.

[50] Fong E, Bargman JM, Chan CT (2007). Cross-sectional comparison of quality of life and illness intrusiveness in patients who are treated with nocturnal home hemodialysis versus peritoneal dialysis. Clin J Am Soc Nephrol.

[51] Roderick P, Nicholson T, Armitage A, Mehta R, Mullee M, Gerard K, Drey N, Feest T, Greenwood R, Lamping D (2005). An evaluation of the costs, effectiveness and quality of renal replacement therapy provision in renal satellite units in England and Wales. Health Technol Assess.

[52] Khatib ST, Hemadneh MK, Hasan SA, Khazneh E, Zyoud SH (2018). Quality of life in hemodialysis diabetic patients: a multicenter cross-sectional study from Palestine. BMC Nephrol.

[53] Javanbakht M, Abolhasani F, Mashayekhi A, Baradaran HR, Jahangiri Noudeh Y (2012). Health related quality of life in patients with type 2 diabetes mellitus in Iran: a national survey. PLoS ONE.

[54] Kang GW, Lee IH, Ahn KS, Lee J, Ji Y, Woo J (2015). Clinical and psychosocial factors predicting health-related quality of life in hemodialysis patients. Hemodial Int.

[55] Acree LS, Longfors J, Fjeldstad AS, Fjeldstad C, Schank B, Nickel KJ, Montgomery PS, Gardner AW (2006). Physical activity is related to quality of life in older adults. Health Qual Life Outcomes.

[56] Merom D, Sinnreich R, Aboudi V, Kark JD, Nassar H (2012). Lifestyle physical activity among urban Palestinians and Israelis: a cross-sectional comparison in the Palestinian-Israeli Jerusalem risk factor study. BMC Public Health.

[57] Drayer RA, Piraino B, Reynolds CF, Houck PR, Mazumdar S, Bernardini J, Shear MK, Rollman BL (2006). Characteristics of depression in hemodialysis patients: symptoms, quality of life and mortality risk. Gen Hosp Psychiatry.

[58] Hemati Z, Alidosti M, Sharifirad G, Kargar M (2013). The relationship between depression and quality of life among hemodialysis patients in Chaharmahal and Bakhtiari province in the year 2011. J Educ Health Promot.

[59] Lopes GB, Matos CM, Leite EB, Martins MT, Martins MS, Silva LF, Robinson BM, Port FK, James SA, Lopes AA (2010). Depression as a potential explanation for gender differences in health-related quality of life among patients on maintenance hemodialysis. Nephron Clin Pract.

[60] Dwyer JT, Larive B, Leung J, Rocco M, Burrowes JD, Chumlea WC, Frydrych A, Kusek JW, Uhlin L (2002). Nutritional status affects quality of life in Hemodialysis (HEMO) Study patients at baseline. J Ren Nutr.

[61] Al Wakeel J, Al Harbi A, Bayoumi M, Al-Suwaida K, Al Ghonaim M, Mishkiry A (2012). Quality of life in hemodialysis and peritoneal dialysis patients in Saudi Arabia. Ann Saudi Med.

[62] Cleemput I, Kesteloot K, Moons P, Vanrenterghem Y, Van Hooff JP, Squifflet JP, De Geest S (2004). The construct and concurrent validity of the EQ-5D in a renal transplant population. Value Health.

[63] Garcia-Llana H, Remor E, Selgas R (2013). Adherence to treatment, emotional state and quality of life in patients with end-stage renal disease undergoing dialysis. Psicothema.

[64] Kao TW, Lai MS, Tsai TJ, Jan CF, Chie WC, Chen WY (2009). Economic, social, and psychological factors associated with health-related quality of life of chronic hemodialysis patients in northern Taiwan: a multicenter study. Artif Organs.

[65] Lopes AA, Bragg-Gresham JL, Goodkin DA, Fukuhara S, Mapes DL, Young EW, Gillespie BW, Akizawa T, Greenwood RN, Andreucci VE (2007). Factors associated with health-related quality of life among hemodialysis patients in the DOPPS. Qual Life Res.

[66] Manns B, Johnson JA, Taub K, Mortis G, Ghali WA, Donaldson C (2003). Quality of life in patients treated with hemodialysis or peritoneal dialysis: what are the important determinants?. Clin Nephrol.

[67] Marinovich S, Lavorato C, Rosa-Diez G, Bisigniano L, Fernandez V, Hansen-Krogh D (2012). The lack of income is associated with reduced survival in chronic haemodialysis. Nefrologia.

[68] Fiebiger W, Mitterbauer C, Oberbauer R (2004). Health-related quality of life outcomes after kidney transplantation. Health Qual Life Outcomes.

[69] Laupacis A, Keown P, Pus N, Krueger H, Ferguson B, Wong C, Muirhead N (1996). A study of the quality of life and cost-utility of renal transplantation. Kidney Int.

[70] Matas AJ, Halbert RJ, Barr ML, Helderman JH, Hricik DE, Pirsch JD, Schenkel FA, Siegal BR, Liu H, Ferguson RM (2002). Life satisfaction and adverse effects in renal transplant recipients: a longitudinal analysis. Clin Transplant.

[71] Bakewell AB, Higgins RM, Edmunds ME (2002). Quality of life in peritoneal dialysis patients: decline over time and association with clinical outcomes. Kidney Int.

[72] Dantas J, Martins MRI (2017). Correlation between pain and quality of life of patients under hemodialysis. Revista Dor.

[73] Devins GM, Armstrong SJ, Mandin H, Paul LC, Hons RB, Burgess ED, Taub K, Schorr S, Letourneau PK, Buckle S (1990). Recurrent pain, illness intrusiveness, and quality of life in end-stage renal disease. Pain.

[74] Kaplan Serin E, Ovayolu N, Ovayolu O (2020). The effect of progressive relaxation exercises on pain, fatigue, and quality of life in dialysis patients. Holist Nurs Pract.

